# A question best addressed in moderation: Religious affiliation and religious/spiritual importance predicting alcohol consumption in Canadians

**DOI:** 10.1371/journal.pone.0340890

**Published:** 2026-04-01

**Authors:** David Speed, Allyson Lamont, Emily Earle, Stanford Yang

**Affiliations:** 1 Department of Psychology, University of New Brunswick, Saint John, New Brunswick, Canada; 2 Department of Psychology, University of New Brunswick, Fredericton, New Brunswick, Canada; Emory University, School of Public Health, UNITED STATES OF AMERICA

## Abstract

One of the strongest findings in the religion/spirituality and health literature addresses the consumption of alcohol: higher levels of religion/spirituality are associated with a greater degree of abstention from, or reduced consumption of, alcohol. However, much of the existing literature assumes the measurement of religious/spiritual importance is independent of the measurement of religious affiliation. Such an assumption has led to a misestimation of the relationship between religion/spirituality and alcohol consumption. Using data from Cycle 30 of the Canadian General Social Survey (*N* > 10,000), we investigated whether religious affiliation *moderated* the relationship between religious/spiritual importance and alcohol consumption. We compared Atheists to 19 other religious groups, with the general expectation that Atheists would report greater alcohol use. In terms of complete alcohol abstention, Protestants who rated religious/spiritual values as very important were more likely to abstain from alcohol completely, while Catholics who rated religious/spiritual values as less important were less likely to abstain from alcohol completely (relative to Atheists). When examining the frequency of alcohol consumption, Atheists were more likely to report higher frequency of alcohol consumption relative to several religious affiliations who also reported higher religious/spiritual importance. Generally, Atheists were statistically similar to religious groups reporting low-to-moderate levels of religious/spiritual importance. The current results suggest that statistical moderation is advantageous in exploring this relationship, and in the literature tying religion/spirituality to alcohol consumption.

## Introduction

Since the 1980s, there has been a sustained interest in the relationship between religion and spirituality (R/S) and health outcomes. Religion is defined as a set of beliefs and behaviours rooted in traditions related to the supernatural, which can take the form of mystical figures, events, or worldviews [1,2]. Spirituality has been described as the search, experience, or expression of “the sacred,” which can be any aspect of life [1,3]. Spirituality has an inclusive meaning because it must capture the variation of individual experiences in the pursuit of the sacred, which would also include religiousness [4–6]. Research in the field of R/S and health has noted that R/S are associated with salutary outcomes [1,7], and adherents report greater physical well-being, fewer mental health symptoms, and greater satisfaction with life [8,9]. While some researchers suggest that R/S are an intrinsic component of health [10,11], more recent research is critical of the R/S-health field for missing, sporadic, or weak effects [12–14]. Specifically, R/S tends to be associated with subjective wellbeing, with little evidence that it is associated with more objective measures of health. A notable exception to this criticism, however, is the relationship between R/S and alcohol. Whether R/S are conceptualized as affiliation [15–18], attending religious services [19–21], prayer [22], or religiosity [23,24], there is a broad consensus that R/S are associated with reduced alcohol consumption. Generally, the protective effects associated with R/S and alcohol consumption are larger than the effects associated with virtually any other health-specific outcome. However, the R/S-alcohol literature suffers from several issues. The first deficiency is one of measurement: nearly all studies assume religious affiliation and religiosity are independent of each other, which is unlikely to be true. The second deficiency is one of benchmarking: while R/S are understood to be salutary, the degree of health differences between religious and nonreligious populations are poorly understood. The current study will address both of these lacunas.

### Alcohol consumption

Alcohol use is prevalent across Canada. In 2019, 76.5% of Canadians aged 15 years and older reported consuming alcohol in the past 12 months [25]. As described by Mirijello et al. [26], the consumption of alcohol has various adverse health effects. In the short-term, alcohol causes generalized impairment of neurocognitive functions and physiological discomfort. At low-to-moderate doses, alcohol can cause impaired psychomotor functioning, memory deficits, and mood alteration, whereas at high doses, it can cause respiratory depression, global neurological impairment, and death [26]. In the long-term, excessive alcohol consumption causes a plethora of adverse health effects, such as increased risk for various types of cancer (e.g., liver, colon) [27–29] and cardiovascular events and diseases [30–32]. Importantly, there are no known health penalties for complete abstention from alcohol [33] and consuming only one alcoholic drink per day appears to be associated with an elevated risk for certain cancers [27].

### Religion/spirituality and alcohol consumption

While a variety of R/S variables are shown to predict alcohol consumption, we focus on religious affiliation and religiosity (i.e., subjective importance of religion) as they are best represented within the literature [2,15]. While religious affiliation can be associated with reduced alcohol consumption, there is substantial nuance in these relationships. Mormons, Pentecostals, and Baptists show greater abstention than Catholics, Lutherans, and Jehovah’s Witnesses [15,19]. Simultaneously, Anglicans, Catholics, and United Church members tend to be comparable to nonreligious groups [18,34]. Generally, there is a *gradient of acceptance* for alcohol where Catholics, Presbyterians, and Lutherans are more tolerant of alcohol relative to Pentecostals, Baptists, and Muslims [19,35]. This gradient implies that grouping individuals into religious versus nonreligious binaries is a poor choice [15,16,36], as religious groups are often dissimilar in attitudes towards alcohol consumption.

Religiosity is also associated with protective effects in relation to alcohol consumption [2,19,24]. When defined as the importance of religious belief in one’s life, religiosity has a small-to-moderate protective effect on alcohol consumption [24,37,38] (although, cf. [39], dissenting), and individuals who self-identify as ‘very religious’ have been found to consume less alcohol compared to those who identify as ‘nonreligious’ [40]. Religiosity, particularly the highest levels of religiosity, are associated with reduced alcohol consumption [36,40]. Additionally, religiosity is a crucial contributor to slowing the progression from initiation to heavy or hazardous alcohol use [41,42].

### Research gap: Mismeasuring religion/spirituality

**Fig 1:**
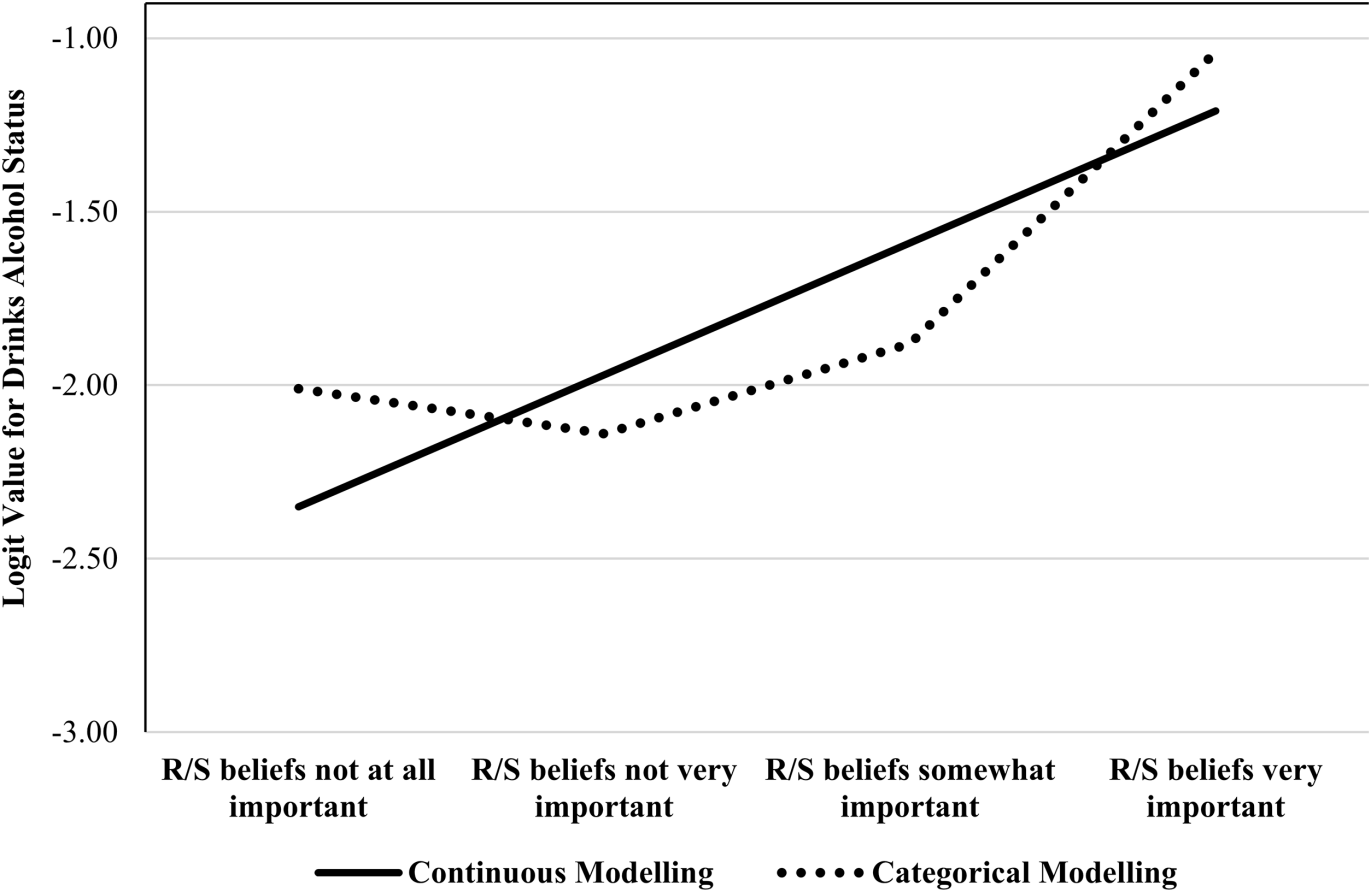
R/S importance Modelled as Continuous and Categorical. *Note*. Data are from the public-use microfile for the Canadian General Social Survey (Cycle 30) and are for illustrative purposes only. Simple logistic regression (0 = Drinks Alcohol; 1 = Does not Drink Alcohol) was used. The slope of R/S importance for the continuous model was, *b* = 0.38, 95% CI [0.34, 0.42], *z* = 19.67, *p <* .001, suggesting each level of R/S Importance was more likely to abstain than the previous ones (every “gap” between the levels of R/S Importance is assumed to be 0.38 units). However, in the categorical model, there is evidence of a quadratic effect, χ^2^(1) = 124.06, *p* < .001, and the difference between ‘No R/S importance’ and ‘Low R/S importance’ was *OR* = 0.88, 95% CI [0.76, 1.01], between ‘No R/S importance’ and ‘Low R/S importance’ was, *OR* = 1.14, 95% CI [1.01, 1.29], and between ‘No R/S importance’ and ‘High R/S importance’ was, *OR* = 2.65, 95% CI [2.36, 2.97]. While both models indicated that R/S Importance was associated with an increased likelihood of alcohol abstention, this was primarily driven by the *highest* level of R/S importance.

Within the R/S-alcohol literature two distinct measurement issues will recur: the assumption that the relationship between R/S and alcohol is linear [16,19,21,24,37,38], and the assumption there is no dependency between religiosity and religious affiliation in their prediction of alcohol consumption [16,35,40,43]. While not every study suffers from both deficiencies [16,40], every study we reviewed experienced at least one. The first issue is that religiosity is often measured using a single term with a multi-item scale (i.e., Likert scales being used as a continuous measurement). This is not intrinsically wrong—provided that increasing religiosity has a smooth relationship with alcohol consumption—but we can see in Fig 1 why this approach may be problematic. Although the continuous model and the categorical model in Fig 1 are based on identical data, it is evident that the models do not strictly agree with each other. The continuous model *underestimates* the likelihood of the ‘No R/S importance’ and ‘High R/S importance’ groups not drinking alcohol and *overestimates* the probability of the ‘Low R/S importance’ and ‘Moderate R/S importance’ groups not drinking alcohol. To make this point explicit, when religiosity is measured as a continuous variable, researchers assume that it has a linear relationship with the outcome. In circumstances where this assumption is unwarranted—like in Fig 1, for instance—then the results are problematic.

The second issue is more subtle and relates to the variability in how specific religious denominations perceive the role of alcohol in society [15,43]. Some religious denominations are more accepting of alcohol than others, suggesting that religiosity may have a unique relationship with alcohol consumption *per* religious denomination. However, if researchers measure religious affiliation and only include a *single* estimate of religiosity in models [16,19,35,39,43], then it is assumed that religiosity has an identical effect across all religious groups. Using Fig 1 as a visual representation, a researcher using a single religiosity term (or a single set of categorical estimates) would assume that the relationship between religiosity and not drinking alcohol is constant and would assume both religious and nonreligious groups report *identical* relationships between religiosity and alcohol consumption. In other words, any individual–be they Protestant or Muslim or religious or nonreligious--would report the same relationship between religiosity and alcohol abstention. However, there is nothing in the literature to suggest that all religious denominations experience uniform benefits from religiosity.

Fortunately, this issue is easily rectified: if religious affiliation is crossed with religiosity, then the resulting interaction terms allow religiosity to vary independently per religious affiliation. This works if religiosity is modelled categorically too, albeit with a greater number of interaction terms being present in the model. The idea that religious affiliation moderates the relationship between religiosity and alcohol consumption is not novel. This idea was initially explored by Marsiglia et al. [23] in a sample of Mexican and Mexican-American youths, examining their use of alcohol, cigarettes, and marijuana. Those results indicated that religiosity had a differential effect for Protestants and Mormons for some of the outcomes. Luczak and colleagues [43] took a similar approach in examining alcohol use behaviours among a sample of Mauritian adults. The findings from this study indicated that religious commitment was only associated with reduced alcohol consumption for participants who perceived their religion as promoting abstention. However, aside from these studies, we could find no recent example of a study exploring moderation effects. This lack of progress is surprising, as Marsiglia et al.’s [23] and Luczak et al.’s [43] results using moderation were promising and demonstrated distinct benefits.

### Research gap: Benchmarking the protective effects of R/S

It is evident that there is *some* relationship between R/S and alcohol consumption, though the extent of the protective effects of R/S are not clearly understood, due in part to the groups that have been compared. Conceptually, if R/S are uniquely health-promoting, then the largest differences in drinking behaviours should be between highly religious individuals and highly nonreligious individuals, as these two groups would represent the ‘two extremes’ of R/S. Atheists are highly nonreligious [14,44,45], so it stands to reason that religious groups should report reduced alcohol consumption relative to atheists generally, and these differences will grow as a function of religiosity. However, only a handful of studies have used atheists as comparators [16,17,39], and each has encountered its own unique difficulties. Chagas et al. [39] found that while high religiosity predicted significantly lower alcohol use than “high atheism/agnosticism”, the effect size was negligible. Gmel and colleagues [16] found that non-believers consumed more alcohol than religious comparators but did not model religiosity. Further, Gmel et al. only used 20-year-olds in their sample, which is a developmental period that tends to see higher rates of alcohol experimentation [25] and secularism [46]. Similarly, Hayward et al. [17] found that atheists were more likely to consume alcohol, but their comparator was an aggregated religious group, which is something that may bias the underlying results [15]. In addition to maximizing the theoretical differences between groups, an added benefit of disaggregating the nonreligious category is that the R/S-health literature is often critiqued for treating the nonreligious as homogeneous rather than heterogeneous [47].

### The current study

To summarize, the R/S-alcohol literature has two notable gaps: 1). The failure to model the interplay between religious affiliation and religiosity; and 2). Understanding the magnitude of the protective influences of R/S. Our research approach addressed both issues. We modelled religious affiliation moderating R/S importance, and we used self-identified atheists as the reference group in all models. This approach allowed us to capture the largest protective effect of R/S *per religious affiliation per level of R/S importance* with respect to alcohol consumption. We have two research questions:

1. Are atheists *less* likely to abstain from alcohol?
2. Do atheists drink alcohol *more* frequently?

## Materials and methods

### Data

All data for the current study were obtained from the master file of the 2016 (Cycle 30) General Social Survey (GSS), a cross-sectional survey designed to cover the ten Canadian provinces [48]. While the GSS is collected annually, different respondents are interviewed each year, and there is no longitudinal component to the data. The focus of the 2016 GSS was work and personal lifestyle, and it was the most recent GSS survey to collect information on alcohol consumption. Data were collected from August 2, 2016, through December 23, 2016, both electronically and by phone. Statistics Canada employees gained informed, verbal consent from respondents. The target population was persons 15 years of age or older living in the Canadian provinces, excluding individuals who were institutionalized. The 2016 GSS stratified individuals geographically and then selected a random sample (without replacement). Cycle 30 had an overall response rate of 50.8%. For the current study, respondents must have reached the age of 20 and answered all covariate questions, religion questions, and the single outcome variable.

We accessed data from Research Data Centres at our institution. The funding for these centres was provided by to the Canadian Research Data Centre Network (CRDCN) from the Social Sciences and Humanities Research Council (SSHRC), the Canadian Institutes for Health Research (CIHR), the Canada Foundation for Innovation (CFI), and Statistics Canada. Although the research and analysis are based on data from Statistics Canada, the opinions expressed do not represent the views of Statistics Canada. Any errors in recoding, analysis, or interpretation are the responsibility of the authors. The authors underwent security screening and were granted permission to use a secure research data centre to access the anonymized data and perform analyses. We followed Statistics Canada’s guidelines for the release of results, but these guidelines did not affect the analyses, only how the descriptive statistics were presented (see Table 1). Please note that Atheists were over a decade younger than the average age of the sample (43.1 years vs. 54.3 years), which is relevant to the eventual results, given that age is negatively correlated with alcohol consumption (see S1 File for age comparisons).

**Table 1:** Descriptive statistics by Religious and Spiritual (R/S) identity.

	*n*	Sex		Frequency of Alcohol Consumption				Education			Age	Income ($10,000)	SRH	SRMH
		Female	Male	<Month	<Week	<4x Week	≥4x Week	≤High School	<Bachelor Degree	≥Bachelor Degree				
All	10,610	51.2%	48.8%	16.8%	28.3%	38.1%	16.7%	35.0%	36.5%	28.5%	54.3/17.5	10.1/19.2	3.5/0.9	3.8/0.9
Atheist	250	34.6%	65.4%	14.8%^†^	22.4%	40.3%	22.5%	25.8%	36.0%	38.2%	43.1/14.9	8.6/5.9	3.5/0.9	3.8/1.0
Catholic														
Nominal	490	45.1%	54.9%	13.6%	25.4%	43.8%	17.2%	31.9%	44.0%	24.2%	46.2/16.5	9.7/15.1	3.6/0.9	3.9/1.0
Cultural	750	48.0%	52.0%	12.0%	23.2%	47.4%	17.4%	37.0%	36.0%	27.0%	49.6/15.0	9.8/9.9	3.6/0.9	3.9/0.9
Practicing	970	56.2%	43.8%	17.4%	26.7%	41.8%	14.2%	41.9%	34.6%	23.5%	54.7/16.0	10.4/36.4	3.5/0.9	3.8/1.0
Committed	500	58.1%	41.9%	23.0%	37.5%	28.2%	11.2%	42.4%	34.3%	23.4%	59.1/17.0	8.6/13.6	3.5/1.0	3.9/1.0
Roman Catholic														
Nominal	350	38.9%	61.1%	17.4%^†^	18.2%	45.0%	19.4%	36.3%	30.4%	33.3%	48.4/16.0	9.3/8.7	3.4/0.9	3.8/1.0
Cultural	610	46.9%	53.1%	14.6%	23.5%	40.4%	21.5%	33.3%	37.9%	28.8%	52.4/17.1	10.2/12.0	3.5/1.0	3.9/0.9
Practicing	1,200	53.3%	46.7%	17.2%	30.2%	38.3%	14.4%	37.6%	39.5%	22.9%	56.7/16.8	10.7/12.0	3.4/1.0	3.8/0.9
Committed	1,000	58.2%	41.8%	24.3%	34.4%	28.5%	12.8%	34.4%	34.1%	31.5%	61.9/17.5	8.9/9.4	3.5/0.9	3.9/0.9
Protestant														
Practicing	290	62.5%	37.5%	16.6%	28.5%	37.0%	17.9%	43.2%	39.3%	17.5%	64.0/16.6	9.1/10.4	3.5/1.0	3.8/0.9
Committed	200	58.9%	41.1%	27.1%	28.3%^†^	30.4%	14.2%^†^	36.1%	39.6%	24.2%^†^	63.9/19.2	8.9/14.7	3.7/0.9	3.9/0.9
Anglican														
Cultural	240	44.7%	55.3%	8.2%^†^	33.0%	36.0%	22.8%^†^	34.4%	40.2%	25.4%	55.3/18.1	14.3/31.9	3.5/0.8	3.8/0.9
Practicing	440	58.8%	41.2%	12.9%	30.7%	38.2%	18.2%	32.5%	37.9%	29.5%	59.9/14.6	10.1/9.2	3.5/1.0	3.7/0.9
Committed	300	65.9%	34.1%	25.2%	28.2%	23.9%	22.7%	33.0%	33.7%	33.3%	64.0/15.3	8.8/9.8	3.4/1.0	3.7/0.9
Christian														
Practicing	300	50.6%	49.4%	14.7%	29.7%	43.3%	12.2%^†^	30.0%	37.9%	32.1%	45.0/14.6	11.1/11.3	3.5/1.0	3.6/1.0
Committed	400	59.1%	40.9%	22.6%	35.2%	30.1%	12.1%^†^	26.1%	35.4%	38.5%	48.2/14.4	11.0/48.9^†^	3.6/1.0	3.8/0.9
United Church														
Cultural	260	46.4%	53.6%	12.0%^†^	29.0%	35.7%	23.3%	34.3%	40.9%	24.9%	57.4/14.0	11.9/14.1	3.6/0.8	3.8/0.9
Practicing	480	62.1%	37.9%	18.0%	26.2%	36.2%	19.6%	37.6%	36.4%	26.0%	61.8/15.0	10.2/19.9	3.4/0.9	3.7/0.9
Committed	270	78.6%	21.4%	23.1%	31.9%	21.7%	23.3%	38.6%	33.5%	27.8%	67.9/14.1	9.0/9.0	3.5/1.0	3.8/0.9
Nonreligious	1,330	42.7%	57.3%	14.2%	29.4%	38.3%	18.1%	31.0%	34.5%	34.5%	44.5/15.3	11.0/12.8	3.5/1.0	3.7/1.0

*Note.* Nominal = R/S beliefs are not at all important. Cultural = R/S beliefs are not very important. Practicing = R/S beliefs are somewhat important. Committed = R/S beliefs are very important. SRH = Self-Rated Health. SRMH = Self-Rated Mental Health. Number of observations is randomly rounded (up or down) to the nearest multiple of 10. Nonreligious group only contains individuals indicating ‘R/S beliefs are not at all important’.

*Note.* Only respondents who indicated that they had drank at least once in their life were included in these analyses.

^†^ Coefficient of variation was moderate (i.e., between 16–33%) interpret point estimates with caution.

In Canada, institutional ethics boards adhere to the Tri-Council Policy regarding the ethical collection of data and the ethical use of secondary data (https://ethics.gc.ca/eng/policy-politique_tcps2-eptc2_2022.html). The Tri-Council Policy statement explicitly exempts the use of secondary data from Statistics Canada. This means we did not require nor did we seek institutional ethics board approval. The lead author accessed the data file between March 2023 and November 2025.

### Measures

#### Alcohol.

The 2016 GSS included a single question on alcohol consumption. Respondents were asked how often they had consumed an alcoholic beverage in the past month (*Every day*, *4–6 times a week*, *1–3 times a week*, *Once or twice in the past month*, *Not in the past month*, *Never had a drink*). While we recognize that these categories are ostensibly ordinal, we would argue that it may be better to think of the final category (i.e., Never had a drink) as a separate *nominal* indicator for our topic. The person has not merely consumed zero drinks in the past month but has consumed zero drinks *across their lifespan*. Research addressing the health implications of alcohol tends to use two standard drinks *per day* as the lowest level of problematic alcohol consumption [25], which makes the categorization of the alcohol outcome somewhat challenging. Including people who never drink in all models will produce genuine *statistical differences*, but less interpretable *practical differences*. For example, finding Group X is more likely to be in the ‘Never Drink’ category rather than the ‘<Monthly Drinking’ category, would have few (if any) real-world health implications. Consequently, we elected to split the alcohol question into two separate questions, one dealing with complete abstention and the other dealing with the frequency of consumption.

***Teetotalling.*** A teetotaller is a person who completely abstains from alcohol. We recoded the alcohol variable such that we measured complete abstention from alcohol (0 = Respondent consumed alcohol at least once; 1 = Respondent has never had a drink). Please note that the legal drinking age in Canada is 18 in some provinces and 19 in other provinces. We used 20 as our age cutoff to ensure there were no boundary cases in which a person would have been motivated to consume alcohol but would not be able to do so due to legal reasons.

***Frequency of alcohol consumption.*** For this variable, we only retained respondents who drank alcohol at least once in their lives. Due to issues with data sparsity across the number of alcohol consumption categories, our categories were recoded (<Monthly, < Weekly, 1–3 days a week; ≥ 4 days a week), with <Monthly being our base category.

#### Covariates.

The 2016 GSS contained a range of sociodemographic variables that were controlled for in all analyses, including age, age^2^, sex (female = reference, male), marital status (married/common-law = reference, widowed/separated/divorced, single), education (high school or less = reference, college or less than bachelor’s, bachelor’s or higher), region (Atlantic = reference, Quebec, Ontario, Prairies, British Columbia), ethnoracial identity (white = reference, Aboriginal, non-Aboriginal/visible minority), total family income (units of $10,000), self-rated health on a 5-point Likert scale, and self-rated mental health on a 5-point Likert scale [49]. All continuous variables were coded such that higher scores indicated greater levels of the outcome.

#### Religion.

Respondents of the 2016 GSS were asked, “What is your religion?”, and were encouraged to specify only one denomination or religion, even if they were not currently a practicing member of the denoted group [49]. Individuals who indicated that they belonged to a general family of religions (e.g., Christian) were asked what specific branch they were affiliated with (e.g., Protestant) but could simply retain their original answer if they so chose. Because religious identification was encouraged *regardless* of whether the individual was a meaningful adherent, we were motivated to parse out the religious categories carefully. Statistics Canada had a 4-point item addressing R/S Importance (“How important are your religious or spiritual beliefs to the way you live your life? Would you say they are...?”), which we used to add context to religious identity. We combined (i.e., crossed) religious affiliation and R/S Importance so that each affiliation had four potential groups: Nominal (*Not at all important*), Cultural (*Not very important*), Practicing (*Somewhat important*), and Committed (*Very important*). Using this “R/S Identity” variable we were able to compare *across* religious affiliations, but also *within* religious affiliations as well. We can explicitly compare alcohol consumption based on an individual’s religious affiliation and their reported level of R/S Importance. In all models Atheists who indicated the lowest level of R/S Importance were our reference group.

While we refer to Nominal Catholics, Committed Protestants, etc. for the remainder of the study, we suggest a degree of caution. These terms are intended as descriptions to assist the reader in differentiating across groups in a convenient fashion. Nominal, Cultural, Practicing, and Committed are narrative descriptors and should not be reified [50]. While a Practicing Catholic and a Practicing Protestant have both identified R/S beliefs as being *Somewhat Important,* how that religious importance manifests to those individuals may not necessarily be equal, comparable, or even similar.

We made two *a priori* decisions regarding R/S Identity. The first decision was a statistical one regarding minimum cell size. Simple power estimation assumed a medium effect between Atheists and other groups, and we calculated that approximately 200 individuals (per group) would provide sufficient power to make meaningful comparisons. Specifically, we could detect an effect of *at least OR* = 2.48 approximately 90% of the time. When comparing the base Atheist group (*n* = 250) to large comparison groups [e.g., Committed Roman Catholics (*n* = 1,000)], our power to detect a medium effect was functionally guaranteed. Given that power analyses are an estimate of the likelihood of statistical significance (which does not convey importance), we would encourage readers to attend to the Odds Ratios and Relative Risk Ratios, as both are metrics of effect size. Please note that by choosing a *meaningful* minimum group size, we were able to discuss null effects (*p* > .05) in a nuanced way. Specifically, when null effects emerged in the teetotalling models, we could reasonably assume that the missed effect was likely smaller than our baseline effect (i.e., *OR* = 2.48). The second decision was a conceptual one regarding heterogeneity of groups. We decided to exclude respondents from the sample who identified as some stripe of nonreligious but *also* reported some degree of R/S Importance. Because there are not defined sets of religious or spiritual beliefs for the nonreligious we were concerned that grouping individuals based on potentially disparate beliefs was problematic. In an ancillary set of analyses however, we relaxed both of these restrictions and the results largely align with the main set of analyses. Ultimately, there were 20 R/S Identities retained in the main set of analyses (see Table 1 for descriptives), and 53 R/S Identities retained in the ancillary analysis.

### Data analysis

All statistical analyses were conducted using Stata 15 software [51] (see S2 File for our syntax). We used a binary logistic regression to assess the teetotaller outcome and a multinomial logistic regression to assess alcohol consumption. We originally sought to use ordinal logistic regression for the alcohol consumption variable, but the model failed to converge (at which stage we explored multinomial logistic regression). The 2016 GSS master file contained both person-level weights, which allowed for corrected point estimates, and bootstrap weights, which allowed for corrected variance estimates. All regression models employed HC1 corrections due to the survey-weighting. We used a nominal *α*-level of.05 for all comparisons (see Limitations). Our analytical approach was as follows:

**Block 1**: An outcome was regressed onto the covariates.
**Block 2**: R/S identity was added to the model, with atheists serving as the base group.

We hypothesized that:

**H1**: Atheists will be *less* likely to be teetotallers.

**H2**: Atheists will be *more* likely to consume alcohol frequently.

## Results

### Teetotalling

**Fig 2:**
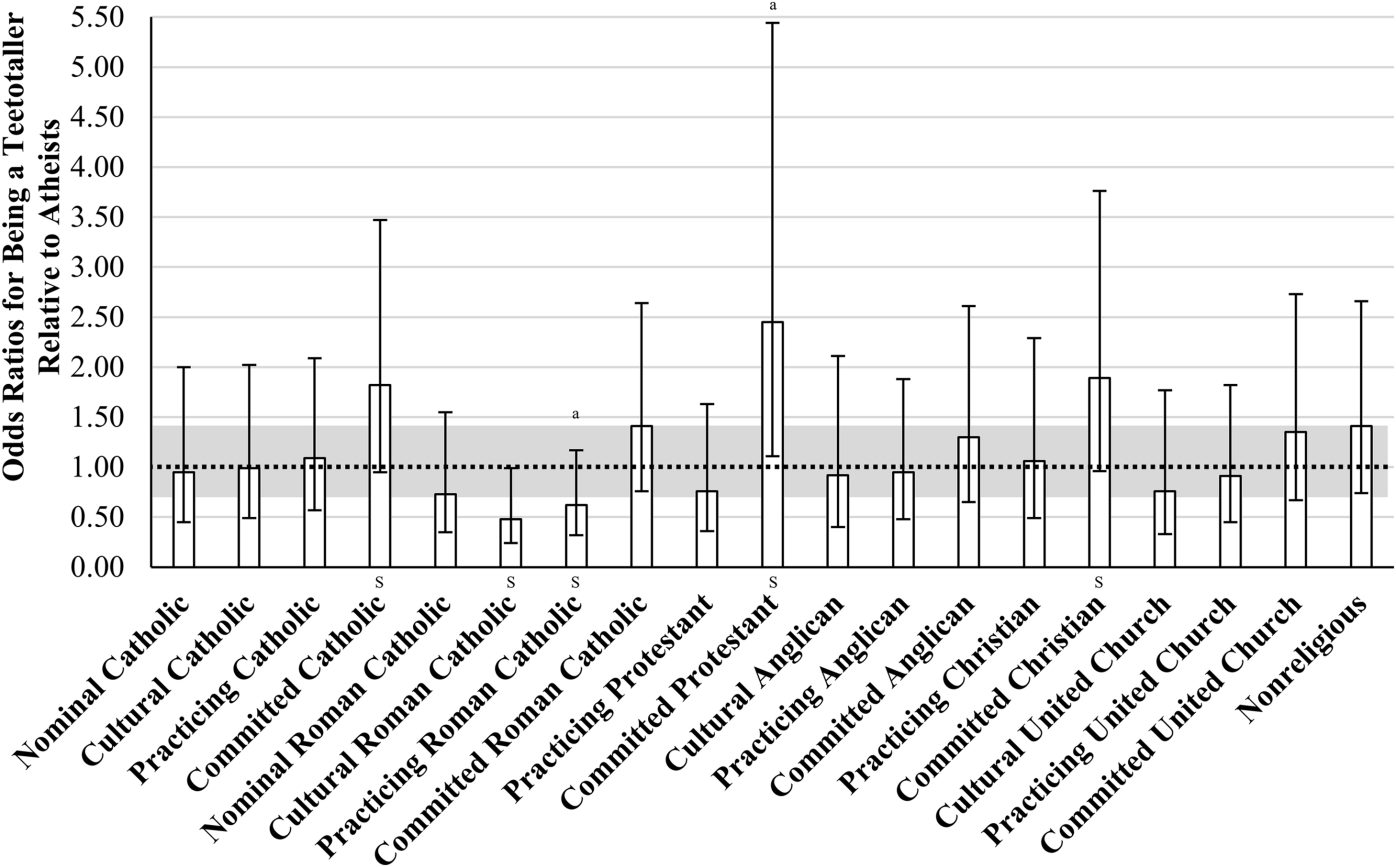
Odds of Being a Teetotaller, Relative to the Atheist Group. *Note*. Estimates are adjusted for sex, region, ethnoracial identity, education, marital status, age, age^2^, household income, self-rated health, self-rated mental health. ORs are relative to the omitted Atheist group (*OR* = 1.00). Grey region is a trivial difference range [0.70 <*OR* < 1.44]. Odds Ratios with 95% CIs are shown. Nominal = R/S beliefs are not at all important. Cultural = R/S beliefs are not very important. Practicing = R/S beliefs are somewhat important. Committed = R/S beliefs are very important. ^S^ = Small Difference; ^M^ = Medium Difference; ^L^ = Large Difference; $OR=\frac{d\pi }{\sqrt{\text{3}}}$ used for converting thresholds. ^a^ *p* < .05.

We regressed Teetotalling onto covariates in Block 1, *F*(16,499) = 22.59, *p* < .001, and added R/S Identity in Block 2, *F*(19,499) = 4.13, *p* < .001. While R/S Identity *was* a significant predictor of Teetotalling behaviours, there was limited support for H1. Relative to Atheists, Committed Catholics were 6% more likely to be teetotallers, M_Diff_ = 0.06, 95% CI [0.00, 0.13], *t* = 2.04, *p* = .041, Commi*tt*ed Protestants were 11% more likely to be teetotallers, M_Diff_ = 0.11, 95% CI [0.01, 0.20], *t* = 2.21, *p* = .027, and Commi*tt*ed Christians were 7% more likely to be teetotallers, M_Diff_ = 0.07, 95% CI [0.00, 0.14], *t* = 2.00, *p* = .046 (see Fig 2). As shown in these resul*t*s, it was the *Committed* members of an affiliation (i.e., high R/S importance) who reported significant differences from Atheists. In terms of effect sizes, Committed Protestants were the most different from Atheists, *OR* = 2.45, 95% CI [1.11, 5.44], *t* = 2.21, *p* = .027, and reported a medium effec*t*. Importantly, other than these groups that have been identified, the remaining 16 groups were similar to Atheists with respect to teetotalling (see Fig 2).

### Frequency of alcohol consumption

**Fig 3:**
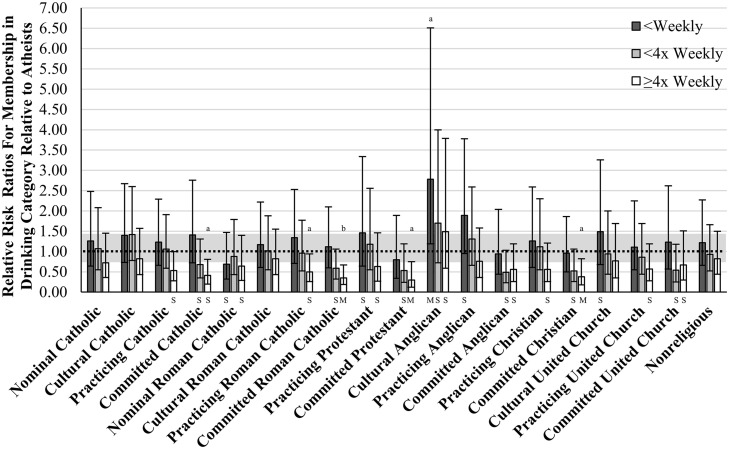
Relative Risk Ratios of Being in a Drinking Category, Relative to Atheist Group. *Note*. Estimates are adjusted for sex, region, racialized identity, education, marital status, age, age2, household income, self-rated health, self-rated mental health. ORs relative to the omitted Atheist group (OR = 1.00). All categories are relative to the ‘<Monthly’ category. Grey region is a trivial difference range [0.70 < RRR < 1.44]. Nominal = R/S beliefs are not at all important. Cultural = R/S beliefs are not very important. Practicing = R/S beliefs are somewhat important. Committed = R/S beliefs are very important. S = Small Difference; M = Medium Difference; L = Large Difference; $RRR=\frac{d\pi }{\sqrt{\text{3}}}$ used for converting thresholds. a p < .05, b p < .01.

We regressed alcohol consumption onto covariates in Block 1, *F*(48,499) = 17.48, *p* < .001, and added R/S Identity in Block 2, *F*(57,499) = 2.12, *p* < .001, with both blocks being significant (see Table 3). Generally, Atheists were fairly similar to non-Atheists for the light drinking categories (<Monthly; < Weekly), but statistical and/or practical differences began to emerge for the heavier drinking categories. Specifically, Atheists were more likely to be in the ≥ 4x Weekly category than were several Committed groups, including Catholics, Roman Catholics, Protestants, and Christians, with varying effect sizes. A weaker pattern of differences was also present for the < 4x Weekly category, something that we will address in the discussion (see Fig 3).

**Table 3:** Multinomial logistic model predicting odds of being in a category different than the ‘Consumes alcohol <monthly’ category.

	Relative Risk Ratios [95% Confidence Intervals]		
	<Weekly	<4 Days Weekly	≥4 Days Weekly
**Constant**	**0.38 [0.14, 1.04]** ^**†**^	**0.10 [0.04, 0.30]** ^***^	**0.01 [0.00, 0.03]** ^***^
Atheist			
Nominal Catholic	1.26 [0.64, 2.48]	1.07 [0.55, 2.08]	0.72 [0.36, 1.45]
Cultural Catholic	1.40 [0.73, 2.67]	1.42 [0.78, 2.60]	0.82 [0.43, 1.57]
Practicing Catholic	1.23 [0.66, 2.29]	1.06 [0.59, 1.91]	0.53 [0.28, 1.00] ^†^
Committed Catholic	1.41 [0.72, 2.76]	0.68 [0.35, 1.31]	0.41 [0.20, 0.81] ^*^
Nominal Roman Catholic	0.69 [0.32, 1.47]	0.88 [0.43, 1.79]	0.64 [0.29, 1.40]
Cultural Roman Catholic	1.17 [0.61, 2.22]	1.02 [0.55, 1.88]	0.82 [0.43, 1.55]
Practicing Roman Catholic	1.34 [0.71, 2.53]	0.96 [0.52, 1.77]	0.50 [0.26, 0.94] ^*^
Committed Roman Catholic	1.12 [0.60, 2.10]	0.59 [0.32, 1.06] ^†^	0.35 [0.19, 0.67] ^**^
Practicing Protestant	1.46 [0.64, 3.34]	1.18 [0.55, 2.56]	0.63 [0.27, 1.46]
Committed Protestant	0.80 [0.34, 1.89]	0.53 [0.24, 1.19]	0.30 [0.12, 0.75] ^*^
Cultural Anglican	2.78 [1.19, 6.51] ^*^	1.70 [0.72, 4.00]	1.49 [0.59, 3.79]
Practicing Anglican	1.89 [0.95, 3.78] ^†^	1.31 [0.66, 2.59]	0.76 [0.36, 1.58]
Committed Anglican	0.94 [0.44, 2.04]	0.49 [0.23, 1.03] ^†^	0.56 [0.26, 1.19]
Practicing Christian	1.26 [0.61, 2.59]	1.12 [0.55, 2.30]	0.56 [0.26, 1.21]
Committed Christian	0.96 [0.50, 1.86]	0.52 [0.26, 1.06] ^†^	0.38 [0.18, 0.82] ^*^
Cultural United Church	1.49 [0.68, 3.26]	0.94 [0.44, 2.00]	0.77 [0.35, 1.69]
Practicing United Church	1.11 [0.55, 2.25]	0.86 [0.44, 1.69]	0.57 [0.28, 1.19]
Committed United Church	1.23 [0.57, 2.62]	0.54 [0.25, 1.18]	0.67 [0.30, 1.51]
Nonreligious	1.22 [0.66, 2.27]	0.93 [0.52, 1.66]	0.82 [0.44, 1.50]

*Note.* For this model, *N* = 10,619. Nominal = R/S beliefs are not at all important. Cultural = R/S beliefs are not very important. Practicing = R/S beliefs are somewhat important. Committed = R/S beliefs are very important. The omitted group for the outcome variable was ‘Less than monthly’ for alcohol consumption. The model controlled for sex, region, racialized identity, education, marital status, age, age squared, family income, self-rated health, and self-rated mental health.

^†^ *p* < .10, * *p* < .05, ** *p* < .01, *** *p* < .001.

### Ancillary analyses

Using the ‘relaxed’ R/S Identity variable, we remodelled the teetotalling outcome (i.e., Table 2) with a greater number of retained groups (i.e., Table 4). First, there was substantive evidence that R/S Minorities were more likely to report not drinking alcohol than Atheists. Specifically, all groups containing Jews, Buddhists, Hindus, Muslims, Sikhs, Mennonites, and Pentecostals were *more likely* to be teetotallers than Atheists. For each of these comparisons the effects were in a practical effect size range. Second, the Atheist base group tended to be in the ‘middle-of-the-pack’ with respect to teetotalling behaviours. While R/S minority groups were much more likely to be teetotallers, many other religious groups with lower levels of R/S Importance were less likely to be teetotallers. These results reinforce Table 2 and Table 3, insofar that Committed R/S Identities were more likely to abstain from alcohol, while other R/S Identities showed a higher degree of variability in those relationships. Third, there was significant variability in how nonreligious groups consumed alcohol. The Atheist and nonreligious groups diverged from each other at times, suggesting that treating each as a separate category was warranted.

**Table 2:** Religious and spiritual identity predicting teetotalling behaviours.

	Odds Ratios [95% Confidence Intervals]	
	Block 1	Block 2
Constant	0.09 [0.04, 0.23] ^***^	0.08 [0.03, 0.24] ^***^
Female		
Male	0.63 [0.53, 0.74] ^***^	0.64 [0.54, 0.76] ^***^
Atlantic		
Quebec	0.57 [0.46, 0.72] ^***^	0.62 [0.48, 0.80] ^***^
Ontario	0.88 [0.72, 1.08]	0.84 [0.69, 1.03] ^†^
Prairies	1.00 [0.81, 1.24]	0.95 [0.77, 1.18]
British Columbia	0.81 [0.61, 1.06]	0.74 [0.55, 0.98] ^*^
White		
Aboriginal	1.28 [0.92, 1.78]	1.28 [0.92, 1.77]
Visible minority	4.10 [3.29, 5.13] ^***^	3.44 [2.72, 4.34] ^***^
High school or less		
College or less than bachelor’s	0.81 [0.68, 0.97] ^*^	0.80 [0.67, 0.97] ^*^
Bachelor’s or higher	0.52 [0.42, 0.65] ^***^	0.51 [0.41, 0.64] ^***^
Married/Common-law		
Widowed/Separated/Divorced	1.08 [0.89, 1.32]	1.07 [0.88, 1.31]
Single	1.28 [1.03, 1.60] ^*^	1.29 [1.03, 1.61] ^*^
Age of Respondent	1.02 [0.99, 1.05]	1.03 [1.00, 1.06] ^†^
Age^2^	1.00 [1.00, 1.00]	1.00 [1.00, 1.00]
Total Family Income ($10,000)	0.96 [0.94, 0.98] ^***^	0.96 [0.95, 0.98] ^***^
Self-Rated Health	0.89 [0.81, 0.97] ^**^	0.87 [0.80, 0.95] ^**^
Self-Rated Mental Health	1.10 [1.00, 1.20] ^*^	1.09 [1.00, 1.19] ^*^
Atheist		
Nominal Catholic		0.95 [0.45, 2.00]
Cultural Catholic		0.99 [0.49, 2.02]
Practicing Catholic		1.09 [0.57, 2.09]
Committed Catholic		1.82 [0.95, 3.47] ^†^
Nominal Roman Catholic		0.73 [0.35, 1.55]
Cultural Roman Catholic		0.48 [0.24, 0.99] ^*^
Practicing Roman Catholic		0.62 [0.32, 1.17]
Committed Roman Catholic		1.41 [0.76, 2.64]
Practicing Protestant		0.76 [0.36, 1.63]
Committed Protestant		2.45 [1.11, 5.44] ^*^
Cultural Anglican		0.92 [0.40, 2.11]
Practicing Anglican		0.95 [0.48, 1.88]
Committed Anglican		1.30 [0.65, 2.61]
Practicing Christian		1.06 [0.49, 2.29]
Committed Christian		1.89 [0.96, 3.76] ^†^
Cultural United Church		0.76 [0.33, 1.77]
Practicing United Church		0.91 [0.45, 1.82]
Committed United Church		1.35 [0.67, 2.73]
Nonreligious		1.41 [0.74, 2.66]

*Note*: For this model, *N* = 12,197. The outcome was coded as 0 = Not a teetotaller and 1 = Teetotaller. Nominal = R/S beliefs are not at all important. Cultural = R/S beliefs are not very important. Practicing = R/S beliefs are somewhat important. Committed = R/S beliefs are very important.

^†^ *p* < .10, * *p* < .05, ** *p* < .01, *** *p* < .001.

**Table 4:** Relaxed religious/spiritual identity predicting teetotalling behaviours.

	Odds Ratios [95% Confidence Intervals]
Constant	0.10 [0.04, 0.25] ^***^
Nominal Atheist	
Cultural Atheist	0.46 [0.13, 1.65] ^S^
Practicing Atheist	1.18 [0.25, 5.53]
Nominal Catholic	0.96 [0.46, 2.02]
Cultural Catholic	1.00 [0.49, 2.05]
Practicing Catholic	1.12 [0.59, 2.15]
Committed Catholic	1.93 [1.01, 3.70] ^* S^
Nominal Roman Catholic	0.74 [0.35, 1.54]
Cultural Roman Catholic	0.49 [0.24, 1.01] ^† S^
Practicing Roman Catholic	0.63 [0.33, 1.22] ^S^
Committed Roman Catholic	1.47 [0.78, 2.78] ^S^
Nominal Protestant	1.24 [0.50, 3.07]
Cultural Protestant	0.99 [0.33, 2.97]
Practicing Protestant	0.78 [0.37, 1.66]
Committed Protestant	2.51 [1.13, 5.61] ^* M^
Nominal Anglican	0.72 [0.29, 1.79]
Cultural Anglican	0.91 [0.39, 2.13]
Practicing Anglican	0.96 [0.48, 1.91]
Committed Anglican	1.33 [0.67, 2.64]
Cultural Baptist	0.54 [0.14, 2.03] ^S^
Practicing Baptist	0.85 [0.29, 2.48]
Committed Baptist	1.86 [0.88, 3.92] ^S^
Nominal Christian	0.56 [0.13, 2.55] ^S^
Cultural Christian	0.57 [0.18, 1.80] ^S^
Practicing Christian	1.06 [0.49, 2.28]
Committed Christian	1.92 [0.97, 3.78] ^† S^
Committed Interdenominational Christian	1.73 [0.59, 5.03] ^S^
Committed Jehovah’s Witness	0.48 [0.18, 1.28] ^S^
Cultural Lutheran	0.97 [0.32, 2.90]
Practicing Lutheran	1.10 [0.46, 2.62]
Committed Lutheran	1.56 [0.59, 4.08] ^S^
Committed Mennonite	8.60 [3.26, 22.72] ^*** L^
Committed Pentecostal	3.44 [1.60, 7.36] ^** L^
Practicing Presbyterian	0.81 [0.32, 2.05]
Committed Presbyterian	1.23 [0.39, 3.92]
Nominal United Church	1.08 [0.32, 3.62]
Cultural United Church	0.75 [0.32, 1.79]
Practicing United Church	0.91 [0.45, 1.82]
Committed United Church	1.35 [0.67, 2.74]
Practicing Jewish	2.71 [0.65, 11.26] ^M^
Practicing Buddhist	1.48 [0.58, 3.78] ^S^
Practicing Hindu	2.65 [1.05, 6.74] ^* M^
Committed Hindu	4.67 [1.89, 11.56] ^*** L^
Practicing Muslim	11.90 [4.83, 29.36] ^*** L^
Committed Muslim	45.42 [17.54, 117.64] ^*** L^
Committed Sikh	8.18 [3.06, 21.81] ^*** L^
Nominal Agnostic	1.91 [0.69, 5.29] ^S^
Cultural Agnostic	0.96 [0.07, 12.82]
Practicing Agnostic	0.48 [0.11, 2.16] ^S^
Nominal Nonreligious	1.39 [0.72, 2.66]
Cultural Nonreligious	1.11 [0.56, 2.21]
Practicing Nonreligious	0.87 [0.44, 1.74]
Committed Nonreligious	1.44 [0.71, 2.93] ^S^

Note. For this model N = 16,410. Nominal = R/S beliefs are not at all important. Cultural = R/S beliefs are not very important. Practicing = R/S beliefs are somewhat important. Committed = R/S beliefs are very important. Outcome was coded 0 = Not a teetotaller and 1 = Teetotaller. The model controlled for sex, region, racialized identity, education, marital status, age, age squared, family income, self-rated health, and self-rated mental health. Any Odds Ratio missing an effect size indicator is within a Trivial range for effect. Boundaries for effect sizes were calculated with $OR=\frac{d\pi }{\sqrt{\text{3}}}.$

^†^ *p* < .10, * *p* < .05, ** *p* < .01, *** *p* < .001.

^S^ Small Effect, ^M^ Medium Effect, ^L^ Large Effect.

## Discussion

The current study explored whether R/S identity predicted teetotalling behaviours and alcohol consumption behaviours in a general sample of Canadians. We hypothesized that atheists would be less likely than all comparators to abstain from drinking, and we hypothesized that atheists would be more likely to drink heavily relative to all comparators. Our results were somewhat mixed. While significant differences *did* emerge for some of the comparisons, the bulk of the comparisons showed *no significant differences* between atheists and non-atheists. Evident in the results, though, is that *highly religious* adherents were more likely to report reduced alcohol consumption relative to atheists, although this pattern was not uniformly present.

### Granularity in the measurement of religion is beneficial

Our specific decision to treat R/S Importance as categorical instead of continuous was theoretically justified and also supported by the patterns of results. If R/S Importance was modelled continuously, then we would expect that each level of increasing R/S Importance would be associated with a uniform change in the likelihood of the dependent variable. However, as is evident in Tables 2 and 3, the relationship between R/S importance and alcohol consumption did not increase or decrease consistently. Instead, there tended to be notable ‘jumps’ associated with moving to the highest level of R/S importance. In Table 2, Nominal, Cultural, and Practicing Catholics all reported a high level of parity with Atheists with respect to teetotalling behaviours (*OR* ≈ 1.00). However, the Committed Catholic group had a notable ‘jump’ in the likelihood of alcohol abstention (*OR* = 1.82). Similarly, Cultural and Practicing Anglicans had near parity with Atheists (*OR* ≈ 0.95), while Committed Anglicans had a ‘jump’ in their likelihood of alcohol abstention (*OR* = 1.30). This pattern was less clear with the ancillary analysis, due in part to the higher degree of error in the point estimates. However, in Table 4 there is evidence that ascending levels of R/S Importance were associated with greater degrees of teetotalling (e.g., Baptists, Lutherans, and generic Christians).

When differences in abstention behaviours emerged, they tended to do so with *Committed* respondents (i.e., individuals reporting the highest level of R/S Importance), and not with respondents with comparatively lower levels of R/S Importance. For the two outcomes, we observed something resembling an *all-or-nothing* relationship between R/S Identity and abstention behaviours, which is present in other studies [36,39]. Frequently, Atheists were *behaviourally indistinguishable* from religious individuals who did not profess the highest level of R/S Importance. While some Practicing members of Hindus and Muslims were more likely to be teetotallers than the Atheist comparator group, these tended to be the exception rather than the rule (Table 4).

Our results were consistent with Marsiglia et al. [23], who found that religious affiliation interacted with religiosity in its prediction of substance use. However, Marsiglia et al. measured religiosity on a 5-point Likert scale and treated it continuously, while we elected to treat our similar R/S Importance variable as categorical, which meant that our models did not assume a linear relationship between the variables. If we had not used interaction terms, we would have implicitly expected that the relationship R/S Importance had with the outcomes did not vary across religious affiliation. That is, Catholics, Roman Catholics, Protestants, Anglicans, Christians, United Church Members, Nones, *and* Atheists would each be expected to report the same relationship with R/S Importance and alcohol consumption. While expecting the same relationship is a good starting point in analyses (it is the initial assumption of null hypothesis significance testing generally), expecting Atheists to report the same relationship as Protestants with respect to R/S Importance is bizarre and not supported in other areas of study [52]. Importantly, our results vindicate both our decision to model R/S Importance as categorical as well as to explore interaction terms.

### Why is there a relationship between R/S and substance use?

A recurring challenge to research on R/S and health is that it is difficult to manipulate religion in a meaningful way to make causal assertions. Consequently, while it is trivial to demonstrate some relationship between R/S and health behaviours, it is difficult to ascertain why this is the case. In the current study, we found that *religious people with high R/S Importance* reported reduced alcohol consumption. Unfortunately, cross-sectional data do not allow testing of theoretical mechanisms, but our immediate discussion serves as a primer on potential reasons this relationship exists. From our perspective, there are two likely candidates to explain why higher levels of R/S are associated with reductions in heavy drinking. The first assumes that belief-behaviour consistency drives the observed pattern of abstention. The second assumes that the *failure* to abstain from alcohol influences how R/S importance was rated. While we explore both, we would stress that the two explanations proffered are not mutually exclusive and that either (or neither) may be responsible for the observed pattern of findings.

### Rules-based abstention

Some religious traditions prohibit or discourage the use of intoxicating substances [53]. Scriptures can influence individuals’ attitudes toward alcohol consumption: for instance, per 1 Corinthians 6:19–20, physical bodies are viewed as temples of the Holy Spirit and are urged to be treated as such [54]. These traditions and interpretations explain why highly religious individuals were less likely to report heavy drinking. The choice to *not* partake in alcohol is *externally* motivated, and religious individuals are ‘following the rules’ regarding the consumption of harmful substances. Some researchers have extended this argument to promote the notion that religious individuals have *internalized* these proscriptions [55]. Rather than simply stating that religious adherents are ‘following the rules’, it is argued that religious adherents have adopted a ‘body-as-a-temple’ belief [56–58]. In short, the human body is a sacred gift from God, and so it is the responsibility of the individual to ensure that they take proper care of their body [56–58]. Case-in-point, Mahoney and colleagues [58] found that individuals who agreed with the statement “My body is a temple of God” were more likely to engage in health-promoting behaviours. However, the ‘body-as-a-temple’ hypothesis seems to be inferred by abstention as opposed to something that is *a priori* predicted. In the literature we reviewed, when abstention failed to materialize, researchers never concluded that the religious and nonreligious were equally likely to perceive their bodies as temples. The selective application of this rationale is concerning as it is functionally confirmation bias.

A minor point with the rules-based abstention is that one need not be religious or spiritual to derive benefits from abstaining from drinking. A staunch Atheist who does not drink alcohol or a devoted Roman Catholic who does not drink alcohol will experience the same health benefits from their teetotalling. If a rules-based abstention explanation accounts for the differences in substance use, it is *incidental*, rather than *intrinsic,* to R/S. Religious or spiritual systems do not need to have prohibitions against intoxicating substances, it is just that many do. Interestingly, some atheist-leaning subcultures have emerged around the rejection of intoxication [59], which would presumably lead to similar health benefits. Consequently, the finding that individuals who have adopted a ‘substance-use-discouraging belief system’ reported ‘reduced consumption of alcohol’ is unsurprising. However, it should also be noted that alcohol consumption is not a black-and-white issue across all religious denominations. While some denominations prohibit alcohol use entirely, for others, alcohol is part of religious traditions, such as drinking wine during the Eucharist [60]. Such nuances highlight the potential ambiguities in relation to alcohol consumption across religious individuals.

### Selecting on the outcome

The second explanation we will discuss is the idea that the behaviours individuals engage in dictate how religious they perceive themselves. How individuals arrive at specific attitudes is well-studied in social psychology. Self-Perception Theory (SPT) [61] suggests that individuals will *infer* their attitudes about a topic based on the behaviour in which they are engaged [62]. SPT originated in the 1960s but has received continual validation since its initial foundations [63,64]. In essence, SPT suggests that individuals evaluate their actions and form attitudes based on them. This theory is germane to the current study for an obvious reason. If an individual is asked how important R/S is to them, they may use a bottom-up approach to assess whether their *behaviour* reflects that R/S is important to them. If a person believes that abstention from drinking is important to a given religious identity, SPT implies that they may consider their drinking behaviours when reporting their religious attitudes. Consequently, a person who is aware that drinking is proscribed, but still drinks alcohol, may infer that they have low R/S importance. By downgrading one’s R/S importance, respondents avoid the associated discrepancy with their own substance use. SPT as an explanation for the findings is intriguing because it also accounts for the pattern of abstention we observed in the data; generally, Nominal, Cultural, and Practicing members reported a similar level of drinking as the Atheist comparators, while Committed members reported reduced levels of drinking. Essentially, it may be that heavier drinkers are ‘opting out’ of the highest R/S Importance category because of their substance use.

## Conclusions

### Limitations

There are several limitations that should be noted for the current study. First, there is a question of its generalizability. For example, despite the cultural similarities between Canada and the United States, research by Wilkins-LaFlamme [65] has indicated that Americans report greater levels of religiosity than Canadians. Moreover, the trajectory of secularization in Canada is unique compared to other countries. Cornelissen [66] examined changes in religiosity in Canada from 1985 to 2019 and found a steady decline in the importance of R/S beliefs (since 2003, when the variable was added to the GSS), religious affiliation, and participation in religious activities. Although some age-related changes were observed, religiosity remained relatively stable within cohorts, and the overall decline appears largely driven by generational shifts toward nonreligion. While similar trends have been observed elsewhere, such as the United States [67], secularization there began later, and religion remains more deeply embedded in American political discourse, societal values, and national identity [68–70]. Furthermore, although Christianity is the majority religion in both countries [66,70], it manifests differently: organizational religiosity is more prominent in Canada, whereas in the United States religion is more individualistic, emphasizing personal belief and a direct relationship with God [71]. Consequently, classifications used in the current study such as Nominal or Cultural identities may not represent equivalent levels of R/S Importance across different regions. Therefore, conclusions about alcohol use in Canada may not apply to other countries.

Second, the present study would have benefited from having a more detailed battery of questions addressing alcohol consumption rather than the single question available in the 2016 GSS. This deficiency provides a logical starting point for future research as the *volume* of alcohol consumed is a better metric of health than the *frequency* at which alcohol is consumed. Specifically, variables addressing binge drinking would add significantly to the literature. In a similar vein, sex as an effect modifier for this relationship makes sense to explore, as females are less likely to consume alcohol frequently and are more likely to be religious.

Third, an explanation we ignored is one of age. The Atheist group was young compared to many other non-Atheist groups. While Statistics Canada does not allow for the release of bivariate statistics, back-of-the-envelope estimates based on Table 1, suggest that Atheists are significantly younger than nearly every other group (see S1 File), and was particularly younger than Committed religious groups (i.e., Cohen’s *d* > 1.00). Given that alcohol consumption is more prevalent in younger individuals [72,73], this may be responsible for the pattern of findings we observed. Essentially, even though we only found modest differences across atheist and highly religious groups, it is still unclear as to whether this is capturing a ‘low-religion vs. high-religion’ pattern, or whether these are simply generational differences (i.e., cohort effects).

Fourth, while our data are representative of Canadians, our exclusion criteria meant that our results were only generalizable to the 20 groups represented in the models. Each of these retained groups represented a single religious affiliation at a distinct level of R/S Importance. By pursuing this approach, we could observe that it was often only the *highest* level of R/S importance that was associated with substantive differences in alcohol consumption, and that differences at lower levels of R/S Importance were muted. While our main results represented more than 15 million Canadians, religious minorities were not included in the main estimates, and the results of the current study cannot be applied to those individuals. However, the results from the ancillary analyses suggest that Atheists tend to be highly similar to most religious comparators with respect to teetotalling behaviours, but are more likely to consume alcohol than R/S minorities (e.g., Muslims, Hindus, Pentecostals).

Fifth, it is necessary to recognize the large number of comparisons in both models. The likelihood of making at least one Type I error was approximately 62% in the teetotaller model (Table 2), approximately 100% in the drinking frequency model (Table 3), and approximately 100% in the teetotaller model with the ‘relaxed’ R/S Identity variable (Table 4). Concerned readers could interpret the results using an α-level of.001 for the comparisons, and this would provide a Familywise-Error Rate of 3% for Table 2, a Familywise-Error Rate of 5.5% for Table 3, and a Familywise-Error Rate of 5.0% for Table 4. If readers elect to do so, we would note that none of the comparisons in either Table 2 nor Table 3 reach conventional significance levels, suggesting that Atheists are quite similar to non-Atheists when multiple comparisons are addressed.

Finally, the current study does not account for recent changes in religious structure in Canada because of the use of 2016 data. Secularization in Canada has been occurring since the 1960s [74], which means that secular groups may demonstrate a different pattern of behaviours today. However, with these caveats in mind, it is important to reiterate that this study had a large, *representative* sample of secular minorities and Christians and addressed the underlying modelling and benchmarking issues present in the literature.

## Summary

Although there is a large body of literature indicating a protective effect of R/S against alcohol consumption, the present findings suggest that this relationship is more nuanced than previously modelled. By including interactions between religious affiliation and R/S importance, our findings provide a deeper understanding of how R/S predicts both abstention and alcohol consumption frequency. Specifically, our results showed that only high R/S Importance groups were more likely to abstain from alcohol than atheists, whereas the low R/S Importance groups were statistically indistinguishable from atheists. These results highlight the importance of considering not only religious affiliation, which may serve as a guide for alcohol-related beliefs and expectancies, but also the level of R/S importance, which may reflect the degree to which an individual’s beliefs would be guided by a particular affiliation (or lack thereof). As secularization continues to rise in Canada, understanding how R/S identity shapes health behaviours is important for anticipating and addressing public health trends.

## Supporting information

S1 File Supplementary age comparisons by Religious and Spiritual Identity. These are crude age comparisons across Religious and Spiritual Identities.
(TXT)

S2 File Syntax File for Analyses. This is the syntax file for the main and supplementary analyses.
(DO)
